# Retrospective epidemiological study for the characterization of community- acquired pneumonia and pneumococcal pneumonia in adults in a well-defined area of Badalona (Barcelona, Spain)

**DOI:** 10.1186/1471-2334-12-283

**Published:** 2012-11-01

**Authors:** Antoni Sicras-Mainar, Jordi Ibáñez-Nolla, Isabel Cifuentes, Pablo Guijarro, Ruth Navarro-Artieda, Lorenzo Aguilar

**Affiliations:** 1 Planning Management Department, Dirección de Planificación y Desarrollo Organizativo, Badalona Serveis Assistencials SA, Gaietà Soler, 6-8 entlo, 08911, Badalona, Barcelona, Spain; 2 Hospital Municipal de Badalona, Badalona, Barcelona, Spain; 3 Medical Department, Pfizer S.L.U., Alcobendas, Madrid, Spain; 4 Pharmacoeconomics Department, Pfizer S.L.U., Alcobendas, Madrid, Spain; 5 Medical Documentation Department, Hospital Germans Trias i Pujol, Badalona, Barcelona, Spain; 6 Microbiology Department, School of Medicine, Universidad Complutense, Madrid, Spain

**Keywords:** Community-acquired pneumonia, *Streptococcus pneumoniae*, Primary care setting, Hospital setting, Resource utilization, Health costs

## Abstract

**Background:**

Community-acquired pneumonia (CAP) has large impact on direct healthcare costs, especially those derived from hospitalization. This study determines impact, clinical characteristics, outcome and economic consequences of CAP in the adult (≥18 years) population attended in 6 primary-care centers and 2 hospitals in Badalona (Spain) over a two-year period.

**Methods:**

Medical records were identified by codes from the International Classification of Diseases in databases (January 1^st^ 2008-December 31^st^ 2009).

**Results:**

A total of 581 patients with CAP (55.6% males, mean age 57.5 years) were identified. Prevalence: 0.64% (95% CI: 0.5%-0.7%); annual incidence: 3.0 cases/1,000 inhabitants (95% CI: 0.2-0.5). Up to 241 (41.5%) required hospitalization. Hospital admission was associated (p<0.002) with liver disease (OR=5.9), stroke (OR=3.6), dementia (OR=3.5), COPD (OR=2.9), diabetes mellitus (OR=1.9) and age (OR=1.1 per year). Length of stay (4.4±0.3 days) was associated with PSI score (β=0.195), in turn associated with age (r=0.827) and Charlson index (r=0.497). Microbiological tests were performed in all inpatients but only in 35% outpatients. Among patients with microbiological tests, results were positive in 51.7%, and among them, *S pneumoniae* was identified in 57.5% cases. Time to recovery was 29.9±17.2 days. Up to 7.5% inpatients presented complications, 0.8% required ICU admission and 19.1% readmission. Inhospital mortality rate was 2.5%. Adjusted mean total cost was €2,332.4/inpatient and €698.6/outpatient (p<0.001). Patients with pneumococcal CAP (n=107) showed higher comorbidity and hospitalization (76.6%), higher PSI score, larger time to recovery and higher overall costs among inpatients.

**Conclusions:**

Strategies preventing CAP, thus reducing hospital admissions could likely produce substantial costs savings in addition to the reduction of CAP burden.

## Background

Community-acquired pneumonia (CAP) accounts for 5% to 12% of all cases of adult lower respiratory tract infections managed by general practitioners in the community [1]. In Spain the annual incidence of CAP in adults varies between 1.6 and 1.8 per 1,000 inhabitants [2]. The incidence of CAP is higher in winter, in older males and in patients with risk factors [3-8]. The percentage of adult patients requiring hospitalization is 22-42%, with between 1.2% and 10% of those admitted to hospital managed on an intensive care unit (ICU) [1].

Despite different available diagnostic tests for CAP, only in nearly 50% of CAP patients the etiological agent is identified [7,9-15], *Streptococcus pneumoniae* being the most frequently identified pathogen [2,16,17]. An adequate clinical assessment for patient classification according to severity prediction factors is essential in CAP management in order to determine the most adequate setting for treatment [13,18-20]. Antimicrobial treatment is empirically initiated after assessing severity, etiology and resistance prevalence in the setting [21-25]. The reported mortality varies widely, from less than 1% in the community to over 30% among patients admitted to ICUs [1]. *S. pneumoniae* is responsible for two-thirds of CAP-related deaths [22].

CAP has a large impact on direct healthcare costs, especially those derived from hospitalization [26-28] that can represent up to 90% of the global cost associated with CAP [19]. Nowadays its prevention relies on quitting smoking habits and vaccination against influenza and *S. pneumoniae *[21,29,30].

Few studies in Spain [5,31] have addressed the epidemiology, impact, evolution and costs of CAP patients in daily practice, both at primary care and hospital settings. The aim of this epidemiological study was to determine the impact, clinical characteristics, outcome and economic consequences of CAP in the adult population attended in primary care centers and hospitals in Badalona (Barcelona, Spain) over a period of two years.

## Material and methods

An observational, retrospective and multicenter study using electronic medical records of both outpatients and inpatients was performed in six primary care centers (Badalona Serveis Assistencials S.A.) and two hospitals (H. Germans Trías i Pujol and H. Municipal) in Badalona (Barcelona, Spain). Clinical data, use of resources and associated costs were recorded over a 6-month period from the date of diagnosis. The study was approved by the Clinical Research Ethics Committee of Hospital Germans Trías i Pujol, Badalona.

Codes R81, 480–487 from the International Primary Care Classification (ICPC-2) [32] and code 481 from the International Classification of Diseases (ninth revision, clinical modification; ICD-9-CM) [33] were used for CAP patients identification in the center’s database. Adult patients (≥18 years) with CAP diagnosis confirmed by radiological findings attended from January 1^st^ 2008 to December 31^st^ 2009 at study centers that complied with follow-up visits were included. Patients were excluded if they were suffering from tuberculosis, lung cancer or were from other sanitary areas.

Data recorded included demographic characteristics, diagnosing setting, clinical data, radiological findings, etiological filiations, antimicrobial treatment according to the ATC classification [34], number of days to clinical cure (from onset of symptoms to recovery) and mortality. Previous antimicrobial treatments (previous week), hospital admissions (previous 12 months) and vaccines administered (*S. pneumoniae* and influenza [last year]) were also recorded. In addition, length of hospital stay, admission to intensive care unit (ICU), readmissions (up to 30 days after discharge), in-hospital complications (organ failure, mechanical ventilation), reasons for discharge and mortality (up to 30 days after discharge) were recorded for hospitalized patients.

Severity was assessed using the *Pneumonia Severity Index (PSI)*[18]. Morbidity was assessed by the *Charlson Comorbidity Index*[35] and *the individual casuistics index* obtained from the *Adjusted Clinical Groups (ACGs) system*[36] estimating individual health status and risk for health service use. ACGs with similar mean cost were grouped in resource utilization bands (RUBs) distributing patients according to morbidity in 5 groups: 1: Healthy-users, 2: mild morbidity 3: moderate morbidity, 4: high morbidity and 5: very high morbidity.

Total costs including direct healthcare costs and indirect costs were calculated. Direct healthcare costs included medical visits (primary care, emergency room, specialists), hospital admissions and readmissions, ICU admissions, complementary tests (laboratory, conventional and complementary radiology) and treatments. Indirect costs were those relative to the number of lost working days and were calculated considering the Spanish minimum interprofessional salary. CAP costs for a 6-month period following CAP diagnosis were per-patient identified, calculated and expressed as mean cost per-patient. Table 1 shows direct and indirect unit costs except those corresponding to treatments. Costs of pharmacological treatments were calculated considering retail price of medicines at the time of prescription.

**Table 1:** Unit health resource costs and lost productivity

**Resource**	**Unit cost (€)**
**Health resources**	
Medical visits	
Primary care	22.74
Emergency room	115.23
Specialist	102.36
Hospitalization (one day)	314.61
ICU (one day)	532.92
**Complementary tests**	
Laboratory tests	21.86
Conventional radiology	18.14
Diagnostic/therapeutic tests	36.45
Pharmacological treatments	RPM
**Productivity**	
Cost per lost workday	54.65

Source of health resources: analytical accounting. Values expressed as means in euros.

RPM: Retail Price of Medicines at the time of prescription.

### Statistical analysis

Patients were distributed into two groups according to the treatment setting: outpatients or inpatients. A descriptive univariate analysis was performed. The Kolmogorov-Smirnov test was used to verify the normal distribution. Median times for clinical cure were determined using the Kaplan-Meier survival curve. Bivariate analyses were carried out by Student’s *t*-test, analysis of variance (ANOVA), Chi-square test and Pearson’s correlation coefficient. Two logistic regression analyses were performed, one using as dependent variable “diagnosis/treatment setting” and the other using “readmission”. In addition, a linear regression analysis was performed using “days of hospitalization” as dependent variable. Ambulatory and hospital costs were compared by analysis of covariance (ANCOVA) following Thomson and Barber recommendations [37], with gender, age, RUBs, Charlson index and PSI score as covariates (Bonferroni-adjusted). The statistical significance was set at p<0.05. The SPSSWIN statistical package, version 18, was used for all statistical analyses.

## Results

**Figure 1:**
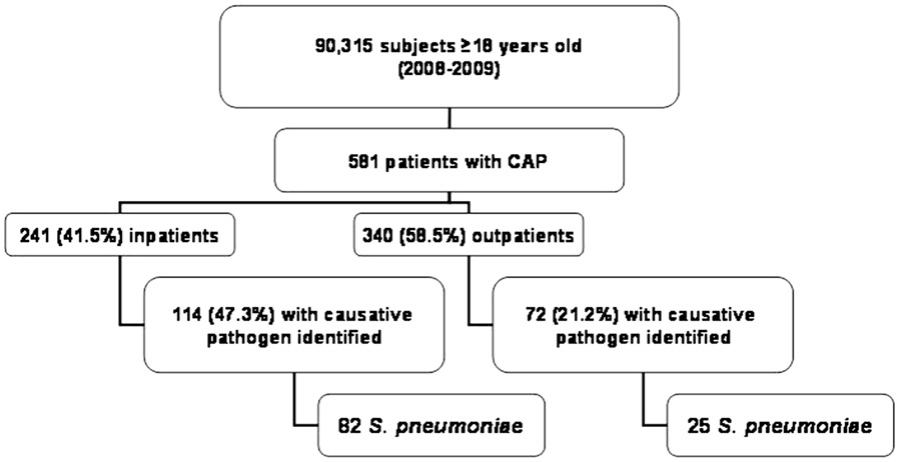
Diagram of patient population.

Among 90,315 subjects ≥18 years belonging to the study centers area, 581 were diagnosed with CAP, 414 (71.3%) of them in the hospital setting (Figure 1). Of the 581 patients identified, 241 (41.5%) required hospitalization and the remaining (340 patients, 58.5%) were followed at their respective primary care center. Prevalence of CAP was 0.64% (95% CI 0.5%-0.7%), annual incidence was 3.0 cases per 1,000 adult inhabitants (95% CI 0.2-0.5) and increased with age: 1.8 for 18–49 years, 3.2 for 50–64 years, 5.1 for 65–74 years and 8.1 for ≥75 years.

A total of 17.4% patients (10.3% for outpatients vs. 27.4% for inpatients, p<0.001) had been hospitalized in the previous 12 months, and 14.5% patients had received an antibiotic course in the previous week (8.8% outpatients vs. 22.4% inpatients, p<0.001). The 23-valent polysaccharide *S. pneumoniae* vaccine had been administered to 23.1% of the overall study population (17.6% outpatients vs. 30.7% inpatients, p<0.001), while the influenza vaccine had been administered to 40.8% patients (30.0% outpatients vs. 56.0% inpatients, p<0.001).

Table 2 shows demographic and clinical data of patients at time of diagnosis. Most participants were males (55.6%), with an overall mean age of 57.5 ± 19.1 years and were not institutionalized (88.3%). Patients that required hospitalization were older (66.6 ± 16.4 years vs. 51.0 ± 18.2 years for outpatients; p<0.001), presented more frequently PSI IV-V (44.4% vs. 7.4% for outpatients; p<0.001) and showed higher morbidity burden (RUB score 3.0 ± 0.7 vs. 2.5 ± 0.9 for outpatients; p<0.001) and Charlson comorbidity index (1.1 ± 0.9 vs. 0.5 ± 1.0 for outpatients; p<0.001). Radiographic evidence of multilobar pneumonia infiltrates was only observed in hospitalized patients (4.1%). In the logistic regression analysis, variables significantly (p<0.002) associated with hospitalization were liver disease (OR=5.9), stroke (OR=3.6), dementia (OR=3.5), COPD (OR=2.9), diabetes mellitus (OR=1.9) and age (OR=1.1 per year of increase).

**Table 2:** Patient characteristics at time of diagnosis

	**Outpatients**^**a**^**N=340**	**Inpatients N=241**	**Total N=581**	**p**
**Age, mean (SD)**	51.0 (18.2)	66.6 (16.4)	57.5 (19.1)	<0.001
18 – 49, %	49.4	15.8	35.5	<0.001
50 – 64,%	25.0	25.3	25.1	
65 – 74, %	12.1	21.2	15.8	
>74, %	13.5	37.8	23.6	
**Gender (male), %**	55.6	55.6	55.6	NS
**Residence, %**				
Private home	87.1	90.0	88.3	<0.001
Health/geriatric institution	12.9	10.0	11.7	
**Pneumonia Severity Index**, mean score	51.4	86.7	66.0	<0.001
I-II, %	79.4	29.9	58.9	<0.001
III, %	13.2	25.7	18.4	
IV-V, %	7.4	44.4	22.7	
**Radiological findings:**				
Unilobar, %	99.4	87.1	94.3	<0,001
Multilobar, %	0.0	4.1	1.7	
Bilateral, %	0.6	8.7	4.0	
**Glycemia**, mg/dL, mean (SD)	109.8 (25.1)	142.0 (53.4)	130.2 (47.7)	<0.001
**Comorbidity, %**				
No. of conditions, mean (SD)	6.0 (3.9)	7.8 (4.2)	6.8 (4.8)	<0.001
Hypertension	29.7	57.7	41.3	<0.001
Diabetes mellitus	9.7	29.9	18.1	<0.001
Dyslipemia	32.6	37.3	34.6	NS
Obesity	25.6	25.3	25.5	NS
Current smoker	25.6	25.3	25.5	NS
History of alcoholism	3.8	5.4	4.5	NS
Ischemic heart disease	7.6	11.2	9.1	NS
Stroke	1.8	13.7	6.7	<0.001
Liver disease	1.2	8.3	4.1	<0.001
Heart failure	3.8	16.2	9.0	<0.001
Renal insufficiency	4.1	14.1	8.3	<0.001
Asthma	9.7	12.4	10.8	NS
COPD^b^	11.8	36.5	22.0	<0.001
Neuropathies	2.6	3.7	3.1	NS
Dementia	1.8	13.3	6.5	<0.001
Depression	14.7	22.4	17.9	0.017
Malignancies	9.4	14.9	11.7	0.041
AIDS	2.1	2.8	2.5	NS
**Charlson comorbid index**, mean (SD)	0.5 (1.0)	1.1 (0.9)	0.8 (0.8)	<0.001
0, %	63.5	42.8	48.7	<0.001
1, %	28.1	37.7	34.9	
2, %	6.6	15.2	12.7	
3, %	0.0	2.4	2.1	
6, %	1.8	1.9	1.5	
**RUB**^**c**^, Mean score (SD)	2.5 (0.9)	3.0 (0.7)	2.7 (0.8)	<0.001
RUB-1, %	13.5	4.6	9.8	<0.001
RUB-2, %	26.5	11.2	20.1	
RUB-3, %	51.2	68.0	58.2	
RUB-4, %	7.6	14.9	10.7	
RUB-5, %	1.2	1.2	1.2	

SD: standard deviation; NS: non significant;

^a^Outpatient: includes patients diagnosed in primary care centres and emergency rooms at hospitals; ^b^COPD: chronic obstructive pulmonary disease; ^c^RUB: Resources utilization bands.

Microbiological tests were performed in all hospitalized patients but only in 119 out of 340 (35.0%) outpatients. Table 3 shows CAP pathogens identified and antimicrobial treatments administered. Among patients with microbiological tests (360/581; 62.0%), results were positive in 51.7% (186/360; 60.5% outpatients vs. 47.3% inpatients, p<0.001). *S pneumoniae* was the most prevalent pathogen identified (57.5%, 107/186): 34.7% outpatients vs. 71.9% inpatients; p<0.001. Fluoroquinolones (prescribed in 52.7% patients) was the most frequent antimicrobial class followed by β-lactams (35.6%), with levofloxacin and amoxicillin/clavulanic acid as the most prescribed compounds. Initial treatment was changed in 7.1% of the patients, mainly due to lack of response.

**Table 3:** CAP pathogens and treatments administered

	**Outpatients**^**a**^**N=340**	**Inpatients N=241**	**Total N=581**	**p**
**Microbiological study**				
Non-studied, n	221	0	221	
Studied, n	119	241	360	
Negative, n	47	127	174	
**Pathogen identified, n**	72	114	186	
*Streptococcus pneumoniae*	25	82	107	<0.001
Influenza virus type A	41	9	50	
*Legionella pneumophila*	5	8	13	
*Haemophilus influenzae*	1	3	4	
*Staphylococcus aureus*	0	3	3	
*Pseudomonas aeruginosa*	0	3	3	
Other pathogens	0	6	6	
**Treatments, %**				
Quinolones	52.9	51.8	52.7	
Penicillins	34.4	37.3	35.6	
Macrolides	5.9	1.7	4.1	
Cephalosporins	1.5	2.5	1.9	
Sulfonamide	0.3	0.8	0.5	
**Combined therapy,** %				
Amoxicillin/Azithromycin	4.1	0.0	2.4	<0.001
Cephalosporin/Azithromycin	0.9	5.8	2.8	
**Change of treatment, %**	5.9	8.7	7.1	NS

^a^Outpatient: includes patients diagnosed in primary care centres and emergency rooms at hospitals.

### Patients’ evolution

Time to recovery was 29.9 ± 17.2 days (27.3 ± 14.5 days for outpatients vs. 33.8 ± 15.7 days for inpatients; p<0.001). Regarding hospitalized patients, 7.5% patients presented some in-hospital complications (4.6% organ failure and 2.9% mechanical ventilation), 0.8% required admission to ICU and 19.1% hospital readmission. In the logistic regression analysis, variables significantly (p<0.05) associated with readmission were diabetes (OR=2.1), number of previous hospitalizations (OR=1.6), Charlson index (OR=1.3), age (OR=1.2 per year of increase) and time to recovery (OR=1.2 per day). Mean length of stay was 4.4 ± 0.3 days. The linear-regression model showed that length of stay was associated with low haematocrit (β= −0.188) and arterial pH (β= −0.161) values and with high PSI score (β= 0.195). A significant (p<0.001) linear correlation was found between the PSI score and age (r= 0.827) and Charlson index (r= 0.497). The reason for discharge was improvement or cure in 90.5% cases, and transfer to other centre in 7.1% patients. The in-hospital mortality rate was 2.5% (95% CI 0.5%-4.5%). None of the ambulatory patients died.

### Resources utilization and associated costs

Use of health resources and lost productivity are given in Table 4. Up to 73.3% of total patients seek medical assistance at the primary care general practitioner’s office and 58.3% at the specialist’s office. Mean number of lost working days was 3.7 ± 11.7 (4.7 ± 12.4 for outpatients vs. 2.1 ± 10.6 for inpatients, p= 0.009), with 14.2% of total patients having some sick leave.

**Table 4:** Use of health resources and lost productivity

	**Outpatients**^**a**^**N=340**		**Inpatients N=241**		**Total N=581**		**p**
	**(%)**	**Mean (SD)**	**(%)**	**Mean (SD)**	**(%)**	**Mean (SD)**	
Medical visits							
Primary care	83.2	2.8 (2.8)	59.3	2.0 (2.5)	73.3	2.5 (2.7)	<0.001
Specialist	48.8	0.9 (2.1)	71.8	1.8 (2.0)	58.3	1.3 (2.1)	<0.001
Emergency room	51.8	0.1 (0.3)	6.5	0.1 (0.2)	32.9	0.1 (0.3)	NS
Test							
Laboratory tests	35.3	0.4 (0.7)	38.6	0.5 (0.7)	36.7	0.4 (0.7)	NS
Conventional radiology	100	0.8 (0.8)	100	0.4 (0.7)	100	0.7 (0.8)	<0.001
Complementary tests	5.9	0.1 (0.3)	7.9	0.1 (0.3)	6.7	0.1 (0.3)	NS
Hospitalizations	---	---	100	4.4 (0.3)	---	---	---
Lost productivity	20.9	4.7 (12.4)	6.2	2.1 (10.6)	14.2	3.7 (11.7)	0.009

NS: non significant; ^a^Outpatient: includes patients diagnosed in primary care centres and emergency rooms at hospitals.

Table 5 shows overall and by-component, per-patient costs. Per-patient mean total expenditure was €1,365.07 (568.48 per outpatient vs. 2,465.65 per inpatient, p<0.001), of which 85.3% (€1,164.49) was due to direct costs and the remaining 14.7% (€201.48) to lost productivity. While direct costs were significantly higher for inpatients (€2,347.05 vs. €326.25 per outpatient, p<0.001), indirect costs were significantly higher for outpatients (€260.23 vs. €118.60 per inpatient, p= 0.009). Healthcare costs were mainly derived from length of hospital stay (60.9%) followed by medical visits (17.2% in total corresponding to 9.7% for specialists, 4.2% for primary care and 3.3% for emergency room visits), pharmacological treatments (5.2%) and diagnostic tests (1.9%).

**Table 5:** Overall and by-component, per-patient costs expressed in Euros

	**Outpatients**^**a**^**N=340**	**Inpatients N=241**	**Total N=581**	**p**	**% Total cost**
Medical visits					
Primary care	65.48	46.42	57.57	<0.001	4.2
Specialist	95.74	185.61	133.02	<0.001	9.7
Emergency room	71.73	7.17	44.95	<0.001	3.3
Test					
Laboratory tests	10.42	10.98	10.65	NS	0.8
Conventional radiology	14.89	8.28	12.15	<0.001	0.9
Complementary tests	2.36	3.48	2.82	NS	0.2
Pharmacological treatments	65.65	78.66	71.05	0.002	5.2
Hospitalizations	0.00	2006.45	832.28		60.9
Hospital admission	0.00	1625.22	674.15		49.3
Readmission	0.00	381.23	158.13		11.6
Direct costs	326.25	2,347.05	1,164.49	<0.001	85.2
Indirect costs	260.23	118.60	201.48	0.009	14.8
**Total costs**	586.48	2,465.65	1,365.97	<0.001	100

^a^Outpatient: includes patients diagnosed in primary care centres and emergency rooms at hospitals.

The adjusted mean total cost per outpatient was €698.5 (direct cost €484.5; indirect cost €214.0) and per inpatient was €2,332.4 (direct cost €2,140.8; indirect cost €191,6). In the multivariate analysis, CAP costs were significantly (p<0.001) associated with readmission (r=0.667), PSI score (r=0.437) and age (r=0.303). Overall, per-patient costs increased with age (<65 years €1,137.96 vs. ≥65 years €1,716.45, p<0.001), due mainly to the increase in direct costs. Among hospitalized patients, significant differences in direct costs were found between patients aged 18–49 years vs. those ≥75 years (€2,151.60 vs. €2,554.84, p= 0.003) while among ambulatory patients no significant differences were found. Patients showing specific comorbidities had significantly (p<0.001) higher hospital-related costs: diabetes mellitus (€3,057.7), stroke (€2,960.2), liver disease (€2,896.6) and COPD (€2,701.9).

### Pn-CAP

Pneumococcal CAP (Pn-CAP) was identified in 82 out of 241 (34.0%) inpatients and in 25 out of 340 (7.4%) outpatients. Prevalence of Pn-CAP was 0.07% and annual incidence was 1.0 cases/1,000 adult inhabitants. Hospitalization rate was 76.6%. Approximately one-third (33.3%) of patients presenting Pn-CAP had been vaccinated with the 23-valent *S pneumoniae* vaccine. Among inpatients with Pn-CAP, comorbidities as COPD (42.7% vs. 33.3%), diabetes mellitus (37.8% vs. 25.8%) and asthma (18.3% vs. 9.4%) were significantly (p<0.05) more frequent than among the remaining inpatients with CAP in the study, without differences in demographic data, treatments administered or in-hospital complications. In addition, mean PSI score (89.3) and time to recovery (36 days, 95% CI 23.7-48.2) were also significantly (p<0.05) higher among hospitalized patients with Pn-CAP. Inpatients with Pn-CAP had a higher overall mean cost (€2,864.7 vs. €2,259.8, p<0.05) and higher direct costs (€2,722.1 vs. €2,153.6, p<0.05), without differences in lost productivity.

## Discussion

Although there is a high variability in published data on annual incidence of CAP, the incidence in the present series (3.0/1,000 adults) is within the range described by others [2,6,13,19,38] but two-times higher than the annual incidence in a previous study in a similar area [37]. Although hospitalization rates depend on the structure of the primary and secondary healthcare system of the studied area, the percentage of patients admitted in hospitals found in our study (41.5%) is in accordance with published rates [7,13,14,36,39] but lower than the percentage described in other studies [37,40] where probably underestimation of ambulatory cases could have occurred. The multivariate analysis performed to investigate variables associated with hospitalization identified liver disease, stroke, dementia, COPD, diabetes mellitus and age as significant variables. Some of them had been previously described as being significantly different between inpatients and outpatients [37]. The length of hospitalization showed linear positive correlation with PSI score, associated in turn with age and Charlson comorbidity index. Of interest is the low percentage of patients requiring ICU admission in our series (0.8%), markedly lower than in other studies [1,37].

No two studies of the etiology of CAP are the same. Differences in frequency of pathogens may be due to healthcare delivery (primary vs. secondary care), hospital and ICU admission practices, population factors (comorbidities, alcoholism…) and study factors [1]. In our series microbiological studies were performed in 62% patients, with great differences in relation to site of care (100% inpatients vs. 35% outpatients), probably because for patients managed in the community microbiological investigations are not recommended routinely [1]. In nearly half of cases with microbiological tests, the etiological agent could not be identified in accordance with results of previous studies [9-15], showing again the need for improving microbiological diagnostic tools for CAP. As expected, *S. pneumoniae* was the most frequent etiological agent among patients with identified pathogen, accounting for approximately one-third of outpatients and two-third of inpatients. Vaccination with the 23-valent pneumococcal vaccine has been reported as cost-effective in individuals aged ≥45 years in our area [41], however, up to 23.1% of patients with Pn-CAP in our series had been previously vaccinated. This finding suggests the need for improving pneumococcal vaccination strategies, an important point since in Spain nonsusceptibility rates to β-lactams and macrolides in *S. pneumoniae* are among the highest in the world [15,42]. However, regardless antimicrobial susceptibility, the link between outcome and serotypes has been described in a published meta-analysis [43]. The fact that levofloxacin was the compound most frequently used as treatment in our series (with macrolides or β-lactam plus macrolides combinations used in <5% cases) contrasts with data from a previous study in our area carried out in 1993–95 where figures were completely different with 65.5% use of macrolides [38], and could be associated with the high non-susceptibility rates to β-lactams and macrolides in *S. pneumoniae* in our country. In the present study, *Legionella pneumophila* was only identified in 7% patients with etiological filiation, without differences in relation to the site of care. This suggests that when establishing empirical antimicrobial therapy in our region, coverage of *Legionella* should be considered even in outpatients.

Pneumonia is the fifth to ninth leading cause of death in developed countries [39,44,45]. Despite mortality of CAP varies depending on the series and site of care, mortality of CAP managed in the community is <1% and from 4% to 10% for hospitalized CAP [1]. In our series, mortality (2.5% for inpatients and 0% for outpatients) was low and length of stay short, probably related to PSI distribution of patients at admission. However, other indices of patient evolution as readmission (19.1%) or time to recovery (29.9 days) were similar or slightly higher than those published [7,37,46,47]. As in a previous study [48], readmissions were associated with comorbidities.

It has been reported that costs of CAP requiring hospital admission are eight-times higher than those managed in the community [37]. This ratio was lower (3.3-times) in our study where the adjusted mean total cost was €698.6 per-outpatient and €2,332.4 per-inpatient. However, costs in the present study were higher than those in previous studies focused on direct hospital costs, both in our country (€1,210 [49], and €1,847 [50]), Germany (€1,201) [51] or Italy (€1,587) [52]. Higher costs in our country are probably related with the more recent study period, the higher rate of readmissions, and mainly with the higher number of analyzed variables, including lost of working days. However, it should be considered that indirect costs in this study could be underestimated since they were calculated based on the minimum interprofessional salary in Spain instead of mean salary amount. In addition, 39.4% patients were ≥65 years, the majority probably retired and thus, without lost of working days. In this sense, the fact that indirect costs were higher in outpatients than inpatients may be related with the significantly higher percentage of patients ≥65 years among inpatients vs. outpatients (59.0% vs. 25.6%). Interestingly, hospitalized patients with Pn-CAP showed significantly higher overall mean costs and direct costs, due to longer time to recovery in relation to significantly higher percentage of comorbidities (COPD, diabetes mellitus and asthma) and PSI score.

Several limitations can be identified in our study making difficult extrapolation of results. It was limited to a specific geographical area, with a specific healthcare system, and costs calculated with local data. In addition, limitations derived from the retrospective nature of the observational study design are also applicable.

## Conclusions

The results of this study show that CAP has a large economic impact derived from the use of healthcare resources for inpatients and both direct and indirect costs for outpatients. Strategies preventing CAP (such as influenza and pneumococcal vaccination of population at risk defined by age and comorbidities), and reducing hospital admission rates (as domiciliary hospitalization programmes) or hospital resources (short-term hospitalization units) could likely produce substantial costs savings in addition to the reduction of CAP burden.

## Abbreviations

CAP: Community-acquired pneumonia; COPD: Chronic obstructive pulmonary disease; ICU: Intensive care unit; Pn-CAP: Pneumococcal CAP; PSI: Pneumonia severity index; RUB: Resource utilization band.

## Competing interests

IC and PG are employees of Pfizer S.L.U, Madrid, Spain.

## Authors’ contributions

Conceived and designed the study: AS-M, IC and PG. Collection of data: AS-M, JI-N, RN-A. Analyzed the data: AS-M, LA. Wrote the paper: AS-M, LA. Reviewed and approved the manuscript: all authors.

## Pre-publication history

The pre-publication history for this paper can be accessed here:

http://www.biomedcentral.com/1471-2334/12/283/prepub
